# Oral contraceptives and colorectal cancer risk: a meta-analysis

**DOI:** 10.1054/bjoc.2000.1622

**Published:** 2001-03

**Authors:** E Fernandez, C La Vecchia, A Balducci, L Chatenoud, S Franceschi, E Negri

**Affiliations:** Institut Català d'Oncologia, L'Hospitalet (Barcelona), Av. Gran Via s/n km 27, 08907, Spain; Istituto di Ricerche Farmacologiche “Mario Negri”, via Eritrea 62, Milan, 20157, Italy; Istituto di Statistica Medica e Biometria, Università degli Studi di Milano, via Venezian 1, Milan, 20133, Italy; International Agency for Research on Cancer, 150 Cours Albert Thomas, Lyon, 69372, France

**Keywords:** colorectal neoplasms, oral contraceptives, female hormones, meta-analysis

## Abstract

Several studies have suggested an inverse association between use of combined oral contraceptives (OC) and the risk of colorectal cancer and here we present a meta-analysis of published studies. Articles considered were epidemiological studies published as full papers in English up to June 2000 that included quantitative information on OC use. The pooled relative risks (RR) of colorectal cancer for ever OC use from the 8 case-control studies was 0.81 (95% confidence interval (CI): 0.69–0.94), and the pooled estimate from the 4 cohort studies was 0.84 (95% CI: 0.72–0.97). The pooled estimate from all studies combined was 0.82 (95% CI: 0.74–0.92), without apparent heterogeneity. Duration of use was not associated with a decrease in risk, but there was some indication that the apparent protection was stronger for women who had used OCs more recently (RR = 0.46; 95% CI: 0.30–0.71). A better understanding of this potential relation may help informed choice of contraception. © 2001 Cancer Research Campaign http://www.bjcancer.com


*British Journal of Cancer* (2001) **84**(5), 722–727
© 2001 Cancer Research Campaign
doi: 10.1054/ bjoc.2000.1622, available online at http://www.idealibrary.com on IDEAL

http://www.bjcancer.com

### Oral contraceptives and colorectal cancer risk: a meta-analysis

**E Fernandez[1,2], C La Vecchia[2,3], A Balducci[2], L Chatenoud[2], S Franceschi[4] and E Negri[2]**

[1]Institut Català d'Oncologia, Av. Gran Via s/n km 2,7, 08907 L'Hospitalet (Barcelona), Spain; [2]Istituto di Ricerche Farmacologiche "Mario Negri", via Eritrea 62, 20157 Milan, Italy; [3]Istituto di Statistica Medica e Biometria, Università degli Studi di Milano, via Venezian, 1, 20133 Milan, Italy; [4]International Agency for Research on Cancer, 150 Cours Albert Thomas, 69372 Lyon, France

**Summary** Several studies have suggested an inverse association between use of combined oral contraceptives (OC) and the risk of colorectal cancer and here we present a meta-analysis of published studies. Articles considered were epidemiological studies published as full papers in English up to June 2000 that included quantitative information on OC use. The pooled relative risks (RR) of colorectal cancer for ever OC use from the 8 case-control studies was 0.81 (95% confidence interval (CI): 0.69–0.94), and the pooled estimate from the 4 cohort studies was 0.84 (95% CI: 0.72–0.97). The pooled estimate from all studies combined was 0.82 (95% CI: 0.74–0.92), without apparent heterogeneity. Duration of use was not associated with a decrease in risk, but there was some indication that the apparent protection was stronger for women who had used OCs more recently (RR = 0.46; 95% CI: 0.30–0.71). A better understanding of this potential relation may help informed choice of contraception. © 2001 Cancer Research Campaign http://www.bjcancer.com

**Keywords:** colorectal neoplasms; oral contraceptives; female hormones; meta-analysis

A role for reproductive and hormonal factors on colorectal carcinogenesis has long been suggested, since an excess of colorectal cancer was reported in nuns (Fraumeni et al, 1969); also, several studies have found an inverse relation between hormone replacement therapy (HRT) and colorectal cancer risk (Herbert-Croteau, 1998). Over the last two decades colorectal cancer mortality has declined more in women than in men in several developed countries (La Vecchia et al, 1998). This may be due to earlier or greater dietary improvements than in men, but exogenous hormones may also play a role (Fernandez et al, 2000a).

Several studies have also provided information on use of combined oral contraceptives (OC) and the risk of colorectal cancer including four cohort studies (IARC Monographs, 1999), of which three showed relative risks (RR) for ever OC use below unity (statistically significant in one). There have been 11 case-control studies, none of which showed significantly elevated risks. The RRs were below unity for 9 studies, and significant in two (IARC Monographs, 1999).

It is therefore of interest to combine all published data on OC and colorectal cancer, to obtain overall and quantitative estimates of the potential association for ever versus never use, and according to duration and recency of use.

### METHODS

Articles considered were epidemiological studies on colorectal cancer published as full papers in English up to June 2000 that included quantitative information on OC use. They were identified by reviewing reference lists in relevant papers, manual and computerized search in Medline and Cancerlit databases, and discussions with colleagues to update the papers included in the IARC Monograph (IARC Monographs, 1999) and a previous review (Franceschi and La Vecchia, 1998). Search strategy included a range of synonyms of neoplasms, tumours, or cancer of colon and/or rectum and of exogenous female hormones, oral contraceptives, oestro-progestins, etc. Studies were eligible only if information had been obtained from each woman, and OCs were distinguishable from hormone replacement and other hormonal therapies. For this reason, we did not include a record-linkage cohort study (Risch and Howe, 1995), which reported no association of OC use with colorectal cancer, and a case-control study (Gerhardsson de Verdier and London, 1992), which showed an inverse association with the use of any female hormone.

A total of 20 papers was reviewed, including 6 from cohort (Chute et al, 1991; Bostick et al, 1994; Martinez et al, 1997; Troisi et al, 1997; Beral et al, 1999; van Wayenburg et al, 2000), and 14 from case-control investigations (Weiss et al, 1981; Potter and McMichael, 1983; Furner et al, 1989; Negri et al, 1989; Kune et al, 1990; Peters et al, 1990; Franceschi et al, 1991; Wu-Williams et al, 1991; Jacobs et al, 1994; Kampman et al, 1994; Fernandez et al, 1996; Kampman et al, 1997; Fernandez et al, 1998; Talamini et al, 1998). Among the cohort studies, only the more recent of the two papers from the Nurses' Health Study (Chute et al, 1991; Martinez et al, 1997) were considered. Among case-control studies, one article (Wu-Williams et al, 1991) included two nonoverlapping study populations from two different geographical areas, and both were included in the meta-analysis. There were 5 articles from 3 case-control studies conducted in Italy: 3 of them (Negri et al, 1989; Franceschi et al, 1991; Fernandez et al, 1996) from two companion studies conducted between 1985 and 1992 in Northern Italy, another (Talamini et al, 1998) from a third study conducted

*Received 11 September 2000*
*Revised 14 November 2000*
*Accepted 14 November 2000*

*Correspondence to:* C La Vecchia

**Table 1** Cohort studies on oral contraceptives and colorectal cancer

| Reference | Country | Population (follow-up) No. of cancers |
|---|---|---|
| Chute et al, 1991; Martinez et al, 1997 | US Nurses' Health Study | 89 448 (12 years) 501 |
| Bostick et al, 1994 | Iowa, US | 35 215 (4 years) 212 |
| Troisi et al, 1997 | US BCDDP | 57 528 (10 years) 95 |
| Beral et al, 1999 | UK RCGP OC Study | 46 000 (25 years) 170 deaths |
| van Wayenburg et al, 2000 | Netherlands | 10 671 (18 years) 95 deaths |

RCGP = Royal College of General Practitioners; BCDDP = Breast Cancer Detection Demonstration Project.

between 1992 and 1996 in 6 Italian areas, and a pooled analysis (Fernandez et al, 1998) that included all studies; the most recent results were routinely included.

For each study, details were extracted on study design, number of subjects (cases and controls or person-years), prevalence of OC use, and control of confounding. Primary analysis concerned the comparison of ever versus never users of OCs, but the influence of duration and recency of use was assessed, wherever possible. In most studies, the combination of cancers of the colon and rectum was, in most instances, the primary outcome, but some concerned only colon cancer, while a few considered colon and rectum separately. We did not assign any quality score to each study, and no studies were excluded a priori for weaknesses of design or data quality.

The measure of effect of interest is the RR for cohort studies, approximated by the odds ratio in case-control studies, and the corresponding statistical significance (95% confidence interval, CI). Summary estimates of the RR were derived using fixed effects models, and heterogeneity was evaluated using a $\chi^2$ test for heterogeneity (Greenland, 1987) and the Galbraith plot (Galbraith, 1988).

Publication bias was evaluated using funnel plots (Thornton and Lee, 2000) and Egger's test (Egger et al, 1997). The RRs and CIs were abstracted from published papers by two of the authors (AB, EF), giving preference to estimates adjusted for multiple confounding factors. When multivariate RRs were not available, these were computed from exposure distribution as given in the articles. There was however little difference between these and the multivariate-adjusted RRs. The weighted average of the estimated RRs was computed by giving each study a weight proportional to its precision (i.e., the inverse of the variance, estimated, when necessary, by calculating the standard errors from the CIs). Summary estimates were calculated for the two types of study separately, as well as in combination.

A graph was given in which a square was plotted for every study, whose centre projection on the underlying scale corresponded to the estimated RR. The area of the square was proportional to the inverse of the variance of the natural logarithm of the RR (Collaborative Group on Hormonal Factors in Breast Cancer, 1996).

### RESULTS

Details of the studies included in the meta-analysis are shown in Table 1 (cohort) and Table 2 (case-control studies).

Figure 1 gives the RRs for ever versus never OC users in the eight case-control and the four cohort studies providing data. The pooled RR from the case-control studies was 0.81 (95% CI: 0.69–0.94).

**Table 2** Case-control studies on oral contraceptives and colorectal cancer

| Reference | Country | Cases:Controls |
|---|---|---|
| Weiss et al, 1981 | Washington, State, US | 143:707 |
| Potter and McMichael, 1983 | Adelaide, Australia | 155:311 |
| Furner et al, 1989 | Chicago, US | 90:208 |
| Kune et al, 1990 | Melbourne, Australia | 190:200 |
| Negri et al, 1989 | | |
| Franceschi et al, 1991 | | |
| Fernandez et al, 1996 | | |
| Talamini et al, 1998 | | |
| Fernandez et al, 1998 | Italy | 1232:2793 |
| Peters et al, 1990 | Los Angeles, US | 327:327 |
| Wu-Williams et al, 1991 | N. America and China | 395:1112 |
| Jacobs et al, 1994 | Seattle, US | 193:194 |
| Kampman et al, 1994 | The Netherlands | 102:123 |
| Kampman et al, 1997 | US, KPMC | 894:1120 |

KPMC = Kaiser Permanente Medical Care.

There was significant heterogeneity among the case-control studies ($\chi^2$ = 26.26, 7 d.f.; $P$ = 0.0005). This, however, was largely due to the study by Weiss et al (1981), which included in the reference group both never users of OCs and users of <1 year. After excluding this study, the summary RR was 0.72 (95% CI: 0.61–0.85), and the heterogeneity was reduced ($\chi^2$ = 12.59, 6 d.f.; $P$ = 0.05). The pooled estimate from cohort studies was 0.84 (95% CI: 0.72–0.97), in the absence of significant heterogeneity ($\chi^2$ = 4.18, 3 d.f.; $P$ = 0.24). The pooled estimate from all studies combined was 0.82 (95% CI: 0.74–0.92). No heterogeneity was present between case-control and cohort studies. No material differences were observed between summary estimates computed from exposure distribution and those derived from multivariate estimates, and hence the fully adjusted estimates were used, whenever available.

For colon cancer (2 cohort and 9 case-control studies, Figure 2), the summary RR was 0.83 (95% CI: 0.74–0.95), without heterogeneity between studies ($P$ = 0.21). For rectal cancer (1 cohort and 5 case-control studies, Figure 3), the summary RR was 0.74 (95% CI: 0.59–0.93), and the heterogeneity between studies was of borderline statistical significance ($P$ = 0.05).

Table 3 gives the summary risk estimates according to different measures of OC use. Duration of use was not related to decrease in risk, since the overall RR of colorectal cancer was 0.78 for short duration of use and 0.85 for long duration. Similarly, no consistent pattern was evident for colon and rectal cancers. Only 2 studies (Fernandez et al, 1998; Beral et al, 1999) included information on recency of use, and there was some indication that the apparent protection was stronger for women who had used OCs more recently (RR = 0.46; 95% CI: 0.30–0.71).

© 2001 Cancer Research Campaign

**Table 3** Oral contraceptives and colorectal cancer: summary RR+ estimates according to duration and recency of use

| | RR+ | 95% CI+ | Studies |
|---|---|---|---|
| Duration of use (based on reported multivariate RR) | | | |
| Colorectal cancer | | | |
| <5 years | 0.78 | 0.64–0.95 | Troisi et al, 1997; Beral et al, 1999; Weiss et al, 1981; Fernandez et al, 1998 |
| ≥5 years | 0.85 | 0.63–1.14 | |
| Colon cancer | | | |
| <5 years | 0.81 | 0.65–1.02 | Chute et al, 1991; Fernandez et al, 1998; Peters et al, 1990; Jacobs et al, 1994 |
| ≥5 years | 0.79 | 0.60–1.05 | |
| Rectal cancer | | | |
| <5 years | 1.05 | 0.68–1.64 | Chute et al, 1991; Fernandez et al, 1998 |
| ≥5 years | 0.94 | 0.59–1.50 | |
| Recency of use (based on reported multivariate RR) | | | |
| Colorectal | | | |
| <10 years | 0.46 | 0.30–0.71 | Beral et al, 1999; Fernandez et al, 1998 |
| ≥10 years | 0.77 | 0.67–0.89 | |

+RR indicates relative risk; CI confidence interval.

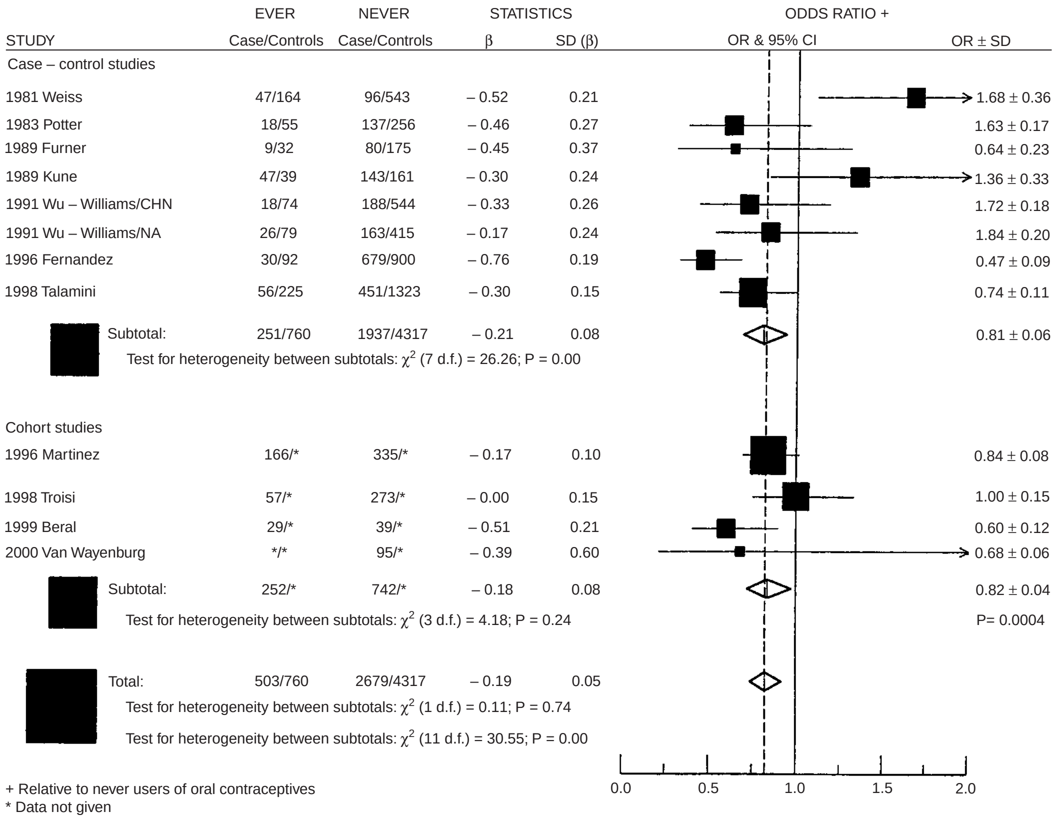

**Figure 1** Summary of relative risk estimates of colorectal cancer for ever vs. never use of oral contraceptives from case-control and cohort studies

### DISCUSSION

This meta-analysis of published studies found a 18% reduction in colorectal cancer risk among ever OC users. This effect was apparently stronger for recent OC use, but there was no duration effect. There was more heterogeneity in case-control than in cohort studies but this was mainly due to one study (Weiss et al, 1981), which was concluded in the years 1976–1977 and did not include a category for never OC users. Apart from one other (Kune et al, 1990), this was the only study to show an increased risk among ever users, and both suffered from low participation rates among cases (about 61%). Since the observed heterogeneity in the meta-analysis

 *© 2001 Cancer Research Campaign*

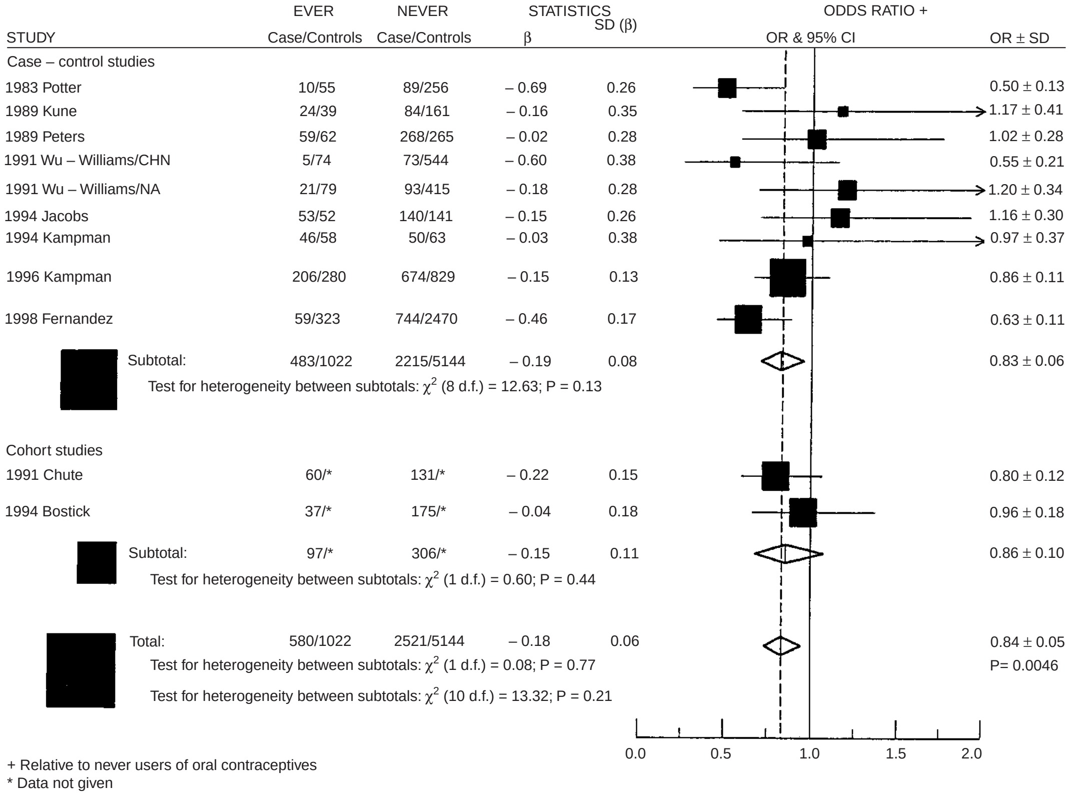

**Figure 2** Summary of relative risk estimates of colon cancer for ever vs. never use of oral contraceptives

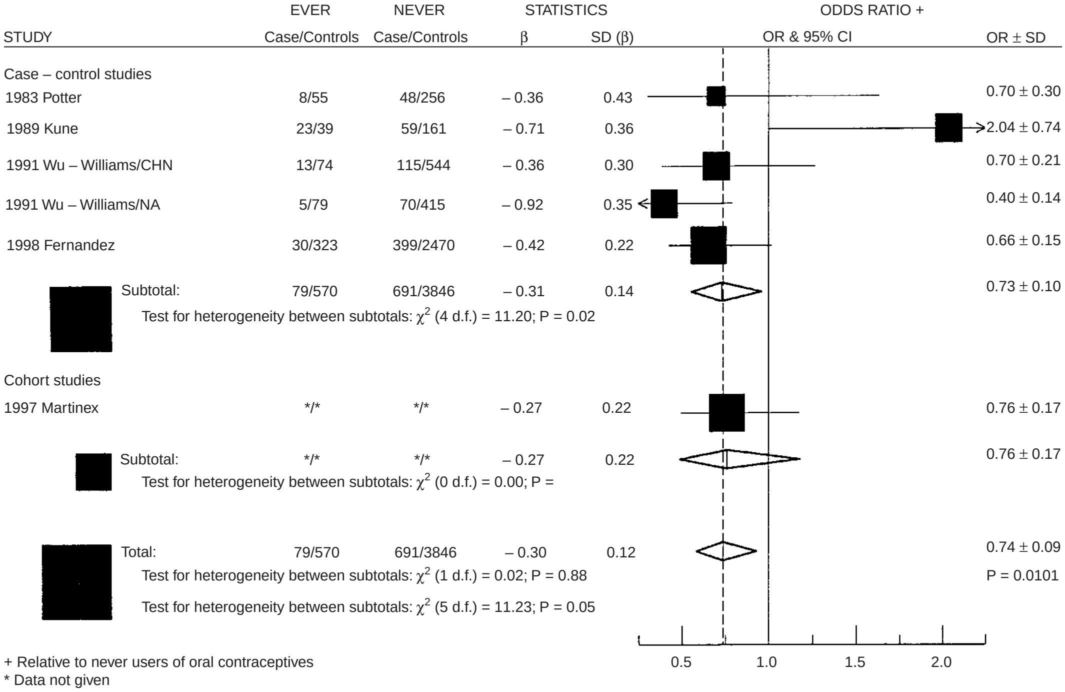

**Figure 3** Summary of relative risk estimates of rectal cancer for ever vs. never use of oral contraceptives was attributable to two of the studies included, we chose to use the fixed effect model rather than the random effect model, which is preferable when the heterogeneity has no simple explanation (Greenland, 1987).

With reference to publication bias, we decided a priori not to search for unpublished data or abstracts, and to exclude studies not based on personal questionnaires. Studies with null results or small sample sizes are less likely to be published (Dickersin and Min,

*© 2001 Cancer Research Campaign*

1993). In the present meta-analyses, however no significant asymmetry was present in the funnel plots, and this can be considered an indicator of the validity of the results.

An important problem concerns allowance for potential confounding factors, including diet, physical activity, socioeconomic indicators and other correlates of colorectal cancer (Potter et al, 1993, 1999). However, the fact that the use of multivariate RRs gave similar pooled estimates to unadjusted ones indicates that the confounding or modifying effect of major considered covariates is unlikely to be substantial.

Most data were collected in the 1980s and 1990s from women with a mean age of 55 to 60 years, and therefore largely refer to OC use between the mid 1960s and the mid 1980s. No information was available on type of OC, but no heterogeneity or systematic trend by calendar year was observed.

Female hormones may protect against colorectal cancer as a result of changes in bile synthesis and secretion, which lead to reduced concentration of bile acids in the colon (McMichael and Potter, 1985). Other biological mechanisms may however be involved, and none of them appears clearly established. Oestrogens inhibit the growth of colon cancer cells in vitro (Lointier et al, 1992), and oestrogen receptors have been identified in normal and neoplastic colon epithelial cells (Thomas et al, 1993). The oestrogen receptor (ER) gene might play a tumour suppressor role, since the hypermethylation of the promotor region of the ER gene results in a reduced expression and deregulated growth in colonic mucosa (Issa et al, 1994). Oestrogens may reduce serum insulin-like growth factor-l (IGF-1) (Campagnoli et al, 1998), a mitogen that has been linked to an increased risk of colorectal cancer (el Atiq et al, 1994; Giovannucci et al, 2000).

Available data therefore suggest that OC use is inversely related to the risk of colorectal cancer. These results are in broad agreement with the descriptive epidemiology of colorectal cancer (dos Santos Silva and Swerdlow, 1996; Fernandez et al, 2000a), with the observation of an inverse relation between HRT and colorectal cancer risk (Herbert-Croteau, 1998; Fernandez et al, 2000b), and with biological hypotheses and experimental findings on the physiologic and molecular pathways of colorectal cancer (McMichael and Potter, 1985; Potter, 1999). A better understanding of this potential relation may help informed choice of contraception (La Vecchia et al, 1996). Some aspects, however, remain undefined, including the risk profile with duration and recency of use, and the possibility of confounding. The issue of causal inference for the observed association is therefore still open to discussion.

### ACKNOWLEDGEMENTS

This work was conducted with the contribution of the Italian Association for Cancer Research, Milan. We are grateful to Anna Schiaffino, Aurelio Tobias and Ivana Garimoldi for technical assistance.

### REFERENCES

Beral V, Hermon C, Clifford K, Hannaford P, Darby S and Reeves G (1999) Mortality in relation to oral contraceptive use: 25-year follow-up of women in the Royal College of General Practitioners' Oral Contraception Study. *Br Med J* **318**: 96–100

Bostick RM, Potter JD, Kushi LH, et al (1994) Sugar, meat, and fat intake, and non-dietary risk factors for colon cancer incidence in lowa women (United States). *Cancer Causes Control* **5**: 38–52

Campagnoli C, Biglia N, Cantamessa C, Lesca L, Lotano MR and Sismondi P (1998) Insulin-like growth factor I (IGF-1) serum level modifications during transdermal estradiol treatment in postmenopausal women: a possible bimodal effect depending on basal IGF-I values. *Gynecol Endocrinol* **12**: 259–266

Chute CG, Willett WC, Colditz GA, Stampfer MJ, Rosner B and Speizer FE (1991) A prospective study of reproductive history and exogenous estrogens on the risk of colorectal cancer in women. *Epidemiology* **2**: 201–207

Collaborative Group on Hormonal Factors in Breast Cancer (1996) Breast cancer and hormonal contraceptives: Collaborative reanalysis of individual data on 53 297 women with breast cancer and 100 239 women without breast cancer from 54 epidemiological studies. *Lancet* **347**: 1713–1727

Dickersin K and Min YI (1993) Publication bias: the problem that won't go away. *Ann N Y Acad Sci* **703**: 135–146

Dos Santos Silva I and Swerdlow AJ (1996) Sex differences in time trends of colorectal cancer in England and Wales: the possible effect of female hormonal factors. *Br J Cancer* **73**: 692–697

Egger M, Davey Smith G, Schneider M and Minder C (1997) Bias in meta-analysis detected by a simple, graphical test. *Br Med J* **315**: 629–634

El Atiq F, Garrouste F, Remacle-Bonnet M, Sastre B and Pommier G (1994) Alterations in serum levels of insulin-like growth factors and insulin-like growth-factor-binding proteins in patients with colorectal cancer. *Int J Cancer* **57**: 491–497

Fernandez E, La Vecchia C, D'Avanzo B, Franceschi S, Negri E and Parazzini F (1996) Oral contraceptives, hormone replacement therapy and the risk of colorectal cancer. *Br J Cancer* **73**: 1431–1435

Fernandez E, La Vecchia C, Franceschi S, Braga C, Talamini R, Negri E and Parazzini F (1998) Oral contraceptive use and risk of colorectal cancer. *Epidemiology* **9**: 295–300

Fernandez E, Bosetti C, La Vecchia C, Levi F, Fioretti F and Negri E (2000a) Sex differences in colorectal cancer mortality in Europe, 1955–1996. *Eur J Cancer Prev* **9**: 99–104

Fernandez E, Franceschi S and La Vecchia C (2000b) Colorectal cancer and hormone replacement therapy: a review of epidemiological studies. *J Br Menop Soc* **6**: 8–14

Franceschi S and La Vecchia C (1998) Oral contraceptives and colorectal tumors. A review of epidemiologic studies. *Contraception* **58**: 335–343

Franceschi S, Bidoli E, Talamini R, Barra S and La Vecchia C (1991) Colorectal cancer in northeastern Italy: reproductive, menstrual and female hormone-related factors. *Eur J Cancer* **27**: 604–608

Fraumeni JF, Lloyd JW, Smith EM and Wagoner JK (1969) Cancer mortality among nuns; role of marital status in etiology of neoplastic disease in women. *J Natl Cancer Inst* **42**: 455–468

Furner SE, Davis FG, Nelson RL and Haenszel W (1989) A case-control study of large bowel cancer and hormone exposure in women. *Cancer Res* **49**: 4936–4940

Galbraith RF (1988) A note on graphical presentation of estimated odds ratios from several clinical trials. *Statist Med* **7**: 889–894

Gerhardsson de Verdier M and London S (1992) Reproductive factors, exogenous female hormones, and colorectal cancer by subsite. *Cancer Causes Control* **3**: 355–360

Giovannucci E, Pollak MN, Platz EA, Willett WC, Stampfer MJ, Majeed N, Colditz GA, Speizer FE and Hankinson SE (2000) A prospective study of plasma insulin-like growth factor-1 and binding protein-3 and risk of colorectal neoplasia in women. *Cancer Epidemiol Biomarkers Prev* **9**: 345–349

Greenland S (1987) Quantitative methods in the review of epidemiologic literature. *Epidemiol Rev* **9**: 1–30

Herbert-Croteau N (1998) A meta-analysis of hormone replacement therapy and colon cancer in women. *Cancer Epidemiol Biomarkers Prev* **7**: 653–659

IARC Monographs on the Evaluation of Carcinogenic Risks to Humans. Vol 72, Hormonal Contraception and Postmenopausal Hormonal Therapy. Lyon: IARC, 1999. pp. 180–186, 294–338

Issa JP, Ottaviano YL, Celano P, Hamilton SR, Davidson EN and Baylin SB (1994) Methylation of the oestrogen receptor CpG islands links ageing and neoplasia in human colon. *Nat Genet* **7**: 536–540

Jacobs EJ, White E and Weiss NS (1994) Exogenous hormones, reproductive history, and colon cancer (Seattle, Washington, USA). *Cancer Causes Control* **5**: 359–366

Kampman E, Bijl AJ, Kok C and van't Veer P (1994) Reproductive and hormonal factors in male and female colon cancer. *Eur J Cancer Prev* **3**: 329–336

Kampman E, Potter JD, Slattery ML, Caan BJ and Edward S (1997) Hormone replacement therapy, reproductive history, and colon cancer: a multicenter, case-control study in the United States. *Cancer Causes Control* **8**: 146–158

 © 2001 Cancer Research Campaign

Kune GA, Kune S and Watson LF (1990) Oral contraceptive use does not protect against large bowel cancer. *Contraception* **41**: 19–25
La Vecchia C, Tavani A, Franceschi S and Parazzini F (1996) Oral contraceptives and cancer. A review of the evidence. *Drug Safety* **14**: 260–272
La Vecchia C, Negri E, Levi F, Decarli A and Boyle P (1998) Cancer mortality in Europe: effects of age, cohort of birth and period of death. *Eur J Cancer* **34**: 118–141
Lointier P, Wildrick DM and Boman BM (1992) The effects of steroid hormones on a human colon cancer cell line in vitro. *Anticancer Res* **12**: 1327–1330
Martinez ME, Grodstein F, Giovannucci E, et al (1997) A prospective study of reproductive factors, oral contraceptive use, and risk of colorectal cancer. *Cancer Epidemiol Biomarkers Prev* **6**: 1–5
McMichael AJ and Potter J (1985) Host factors in carcinogenesis: certain bile-acid metabolic profiles that selectively increase the risk of proximal colon cancer. *J Natl Cancer Inst* **75**: 185–191
Negri E, La Vecchia C, Parazzini F, et al (1989) Reproductive and menstrual factors and risk of colorectal cancer. *Cancer Res* **49**: 7158–7161
Peters RK, Pike MC, Chang WWL and Mack TM (1990) Reproductive factors and colon cancers. *Br J Cancer* **61**: 741–748
Potter JD (1999) Colorectal cancer: molecules and populations. *J Natl Cancer Inst* **91**: 916–932
Potter JD and McMichael AJ (1983) Large bowel cancer in women in relation to reproductive and hormonal factors: a case-control study. *J Natl Cancer Inst* **71**: 703–709
Potter JD, Slattery ML, Bostick RM and Gapstur SM (1993) Colon cancer: a review of the epidemiology. *Epidemiol Rev* **15**: 499–545
Risch HA and Howe GR (1995) Menopausal hormone use and colorectal cancer in Saskatchewan: A record linkage cohort study. *Cancer Epidemiol Biomarkers Prev* **4**: 21–28
Talamini R, Franceschi S, Dal Maso L, Negri E, Conti E, Filiberti R, Montella M, Nanni O and La Vecchia C (1998) The influence of reproductive and hormonal factors on the risk of colon and rectal cancer in women. *Eur J Cancer* **34**: 1070–1076
Thomas ML, Xu X, Norfleet AM and Watson CS (1993) The presence of functional estrogen receptors in intestinal epithelial cells. *Endocrinology* **132**: 426–430
Thornton A and Lee P (2000) Publication bias in meta-analysis: its causes and consequences. *J Clin Epidemiol* **53**: 207–216
Troisi R, Schairer C, Chow W-H, Schatzkin A, Brinton LA and Fraumeni JF Jr (1997) Reproductive factors, oral contraceptive use, and risk of colorectal cancer. *Epidemiology* **8**: 75–79
van Wayenburg CAM, van der Schouw YT, van Noord PAH and Peeters PHM (2000) Age at menopause, body mass index, and the risk of colorectal cancer mortality in the Dutch Diagnostisch Onderzoeck Mammacarcinoom (DOM) cohort. *Epidemiology* **11**: 304–308
Weiss NS, Daling JR and Chow WH (1981) Incidence of cancer of the large bowel in women in relation to reproductive and hormonal factors. *J Natl Cancer Inst* **67**: 57–60
Wu-Williams AH, Lee M, Whittemore AS et al (1991) Reproductive factors and colorectal cancer risk among Chinese females. *Cancer Res* **51**: 2307–2311

*© 2001 Cancer Research Campaign*
